# Recurrent Shoulder Instability With Glenoid Bone Loss: A Case Report on Congruent Arc Latarjet

**DOI:** 10.7759/cureus.94453

**Published:** 2025-10-13

**Authors:** Kashif Memon, Manahil Awan, Shahzad Ahmad, Shenouda Shehata Abdelmesih, Ahmed A Ali

**Affiliations:** 1 Trauma and Orthopedics, Queen Elizabeth Hospital Birmingham, Birmingham, GBR; 2 Surgery, Liaquat National Hospital, Karachi, PAK; 3 Orthopedics and Traumatology, Khoula Hospital, Muscat, OMN; 4 Emergency, Mubarak Al-Kabeer Hospital, Hawally, KWT

**Keywords:** arthroscopy, congruent arc latarjet, glenohumeral dislocation, glenoid bone loss, hill-sachs lesion, recurrent shoulder instability

## Abstract

Recurrent shoulder instability is challenging and is linked to significant bony defects such as glenoid bone loss and Hill-Sachs lesions (HSLs). The Latarjet procedure, particularly its congruent arc modification, provides a dependable solution for complex cases with combined glenoid and humeral bone loss. We report the case of a 23-year-old wrestler with recurrent left shoulder dislocations over seven years, severely impairing his daily activities. Clinically, there was a positive apprehension test and limited external rotation. Imaging showed a large HSL and an inverted pear-shaped glenoid, indicating marked bone loss. Glenoid track (GT) analysis confirmed an off-track lesion with high engagement risk. After diagnostic arthroscopy confirmed the bone loss, an open congruent arc Latarjet (CAL) procedure was performed via a deltopectoral approach. The coracoid graft was fixed with two parallel screws, and postoperative radiology verified proper placement. At three months, the patient regained full, pain-free motion without recurrence or functional limitation. This case highlights the importance of early bone loss recognition, accurate imaging, and meticulous planning. The CAL remains an effective option for restoring stability in high-demand patients.

## Introduction

The shoulder (glenohumeral joint) is a synovial, ball-and-socket, diarthrodial structure between the shallow glenoid and larger hemispherical humeral head [1]. The shoulder is a highly mobile and unstable joint due to a shallow glenoid that only articulates with a small part of the humeral head, which is susceptible to various abnormalities, among which dislocations and instabilities are the most frequent [2]. Shoulder dislocations represent 50% of all major joint dislocations, with anterior dislocation being the most common. The incidence of anterior shoulder instability is 0.08 per 1,000 person-years in the general population, with much higher reported rates for young high-risk men at 3% per year compared to posterior and inferior dislocations [3]. The common provocative tests for the assessment of anterior instability include the load and shift test, the anterior apprehension test, and the relocation sign. A positive Jerk test, Kim test, or push-pull test may suggest posterior instability. Sulcus and Gagey signs can suggest inferior instability or capsular laxity and are useful for evaluating multidirectional instability. The Beighton score for generalized ligamentous laxity should be assessed. A score of over five out of nine should raise the suspicion of ligamentous laxity [4]. Dislocation is frequently associated with anatomical anomalies, including labral tears and rotator cuff injuries. One of the well-recognized complications of recurrent dislocations is the Hill-Sachs lesion (HSL). HSL is categorized as a bony defect of the posterosuperolateral humeral head, often caused by prior episodes of anteroinferior glenohumeral dislocation.

The prevalence of HSLs ranges from 70% to 90% after an anterior shoulder dislocation and may approach up to a 100% incidence rate in patients with recurrent anterior shoulder instability [5]. Various types of HSLs have been described based on size and engagement with the glenoid rim. One of the classifications of HSLs is according to the glenoid track (GT) paradigm by arthroscopic and radiographic measurements [6]. Management of shoulder dislocation and associated lesions includes both conservative and surgical options, chosen based on the patient’s age, activity level, and the extent of bone or soft tissue injury. Conservative measures, such as physiotherapy and activity modification, may suffice for first-time dislocations without significant bone loss, while recurrent instability often requires surgery. Assessment of bone defects using the “on-track/off-track” concept is crucial; off-track HSLs, which risk engagement with the glenoid rim, generally require bony augmentation procedures like the Latarjet. Shoulder stability relies on static and dynamic stabilizers, particularly the inferior and middle glenohumeral ligaments (IGHL and MGHL), which act as a “hammock” supporting the humeral head. Clinical examination tests, including apprehension and relocation maneuvers, help identify the direction and severity of instability [7]. This case report highlights the presentation, diagnostic challenges, and surgical management of recurrent shoulder instability with significant glenoid bone loss, emphasizing the effectiveness of the congruent arc Latarjet (CAL) procedure in restoring stability and function.

## Case presentation

**Figure 1:**
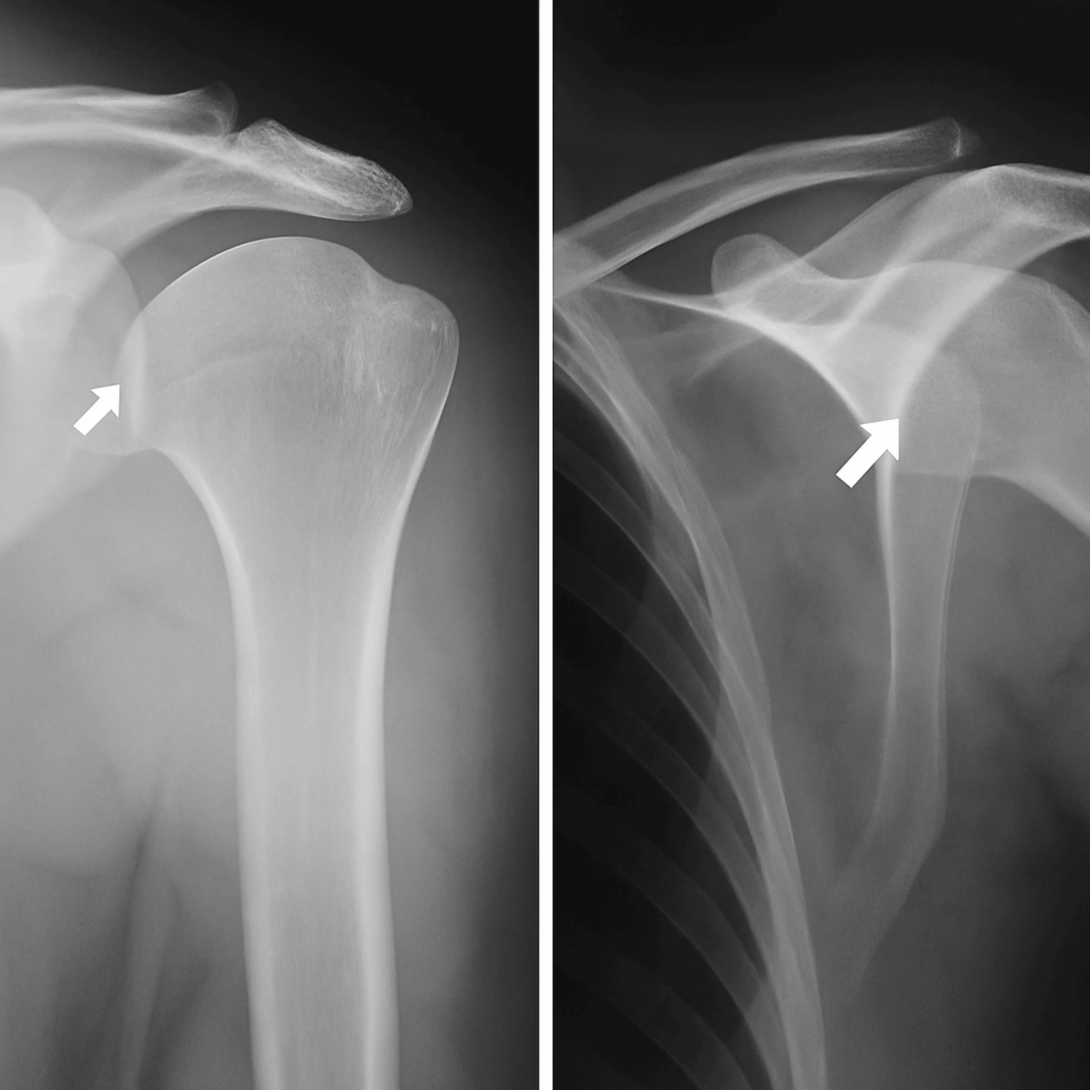
Large Hill-Sachs lesion on the posterosuperior aspect of the humerus X-ray of the left shoulder reveals a wedge-shaped, large Hill-Sachs lesion on the posterosuperior aspect of the humeral head (arrow).

**Figure 2:**
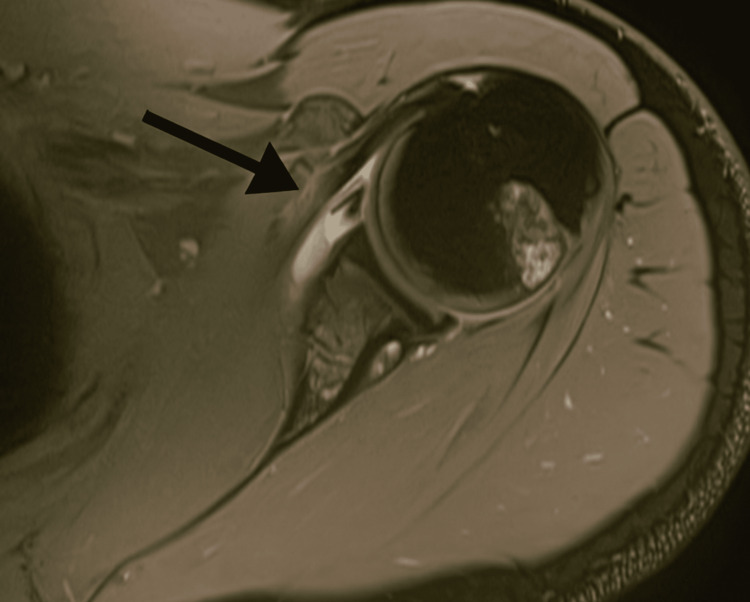
MRI showed normal orientation of the humeral head with a very large Hill-Sachs lesion Axial MRI of the shoulder demonstrating a Hill-Sachs lesion (arrow), showing a cortical depression in the posterolateral aspect of the humeral head.

In January 2022, a 23-year-old male wrestler with no known comorbidities or allergies presented to the outpatient department (OPD) with complaints of recurrent left shoulder dislocations, eight times over seven years, and difficulty in performing overhead activities. The patient reported episodes of shoulder dislocation even during routine tasks such as combing his hair. On examination, he was a young male of average height and build with a normal shoulder contour. The range of motion of the left shoulder was preserved in comparison to the right shoulder, but internal rotation of the left shoulder elicited pain, and external rotation was limited to 70 degrees. The apprehension test was positive, with audible and palpable clicking during movement. Beighton’s score was within the normal range, and the sulcus sign was negative. Baseline workup was suggested in OPD, including an X-ray of the left shoulder, and follow-up was advised (Table 1). An X-ray of the left shoulder revealed a large HSL on the posterosuperior aspect of the humeral head (Figure 1). Magnetic resonance imaging (MRI) was planned to further delineate the pathology. MRI revealed normal orientation of the humeral head with a very large Hill-Sachs defect with an inverted pear-shaped glenoid, indicating a bipolar lesion involving both the humeral head and glenoid (Figure 2). Glenoid track (GT) analysis was performed (GT = 17.9 mm, Hill-Sachs = 18.8 mm), confirming an off-track lesion with significant risk of engagement and recurrent instability.

**Table 1:** Baseline workup Hb: hemoglobin; WBC: white blood cells; PLT: platelets; PT: prothrombin time; aPTT: activated partial thromboplastin time; INR: international normalized ratio; AST (SGOT): aspartate aminotransferase (serum glutamic-oxaloacetic transaminase); ALP: alkaline phosphatase; BUN: blood urea nitrogen; Cr: creatinine; Na⁺: sodium; K⁺: potassium; Cl⁻: chloride.

Lab Test	Patient’s Value	Normal Value	Unit / Notes
Hemoglobin	11	12 – 16 (female) 13 – 17 (male)	g/dL
White Blood Cells (WBC)	7600	4,000 – 11,000	cells/µL
Platelets	20300	150,000 – 400,000	cells/µL
PT	12.3	11 – 13.5	seconds
aPTT	28.2	25 – 35	seconds
INR	0.12	0.8 – 1.2	Ratio
AST (SGOT)	24	10 – 40	U/L
ALP	56	44 – 147	U/L
Urea (BUN)	18	7 – 20	mg/dL
Creatinine	0.9	0.6 – 1.3	mg/dL
Sodium (Na⁺)	136	135 – 145	mmol/L
Potassium (K⁺)	4.2	3.5 – 5.0	mmol/L
Chloride (Cl⁻)	102	96 – 106	mmol/L

**Figure 3:**
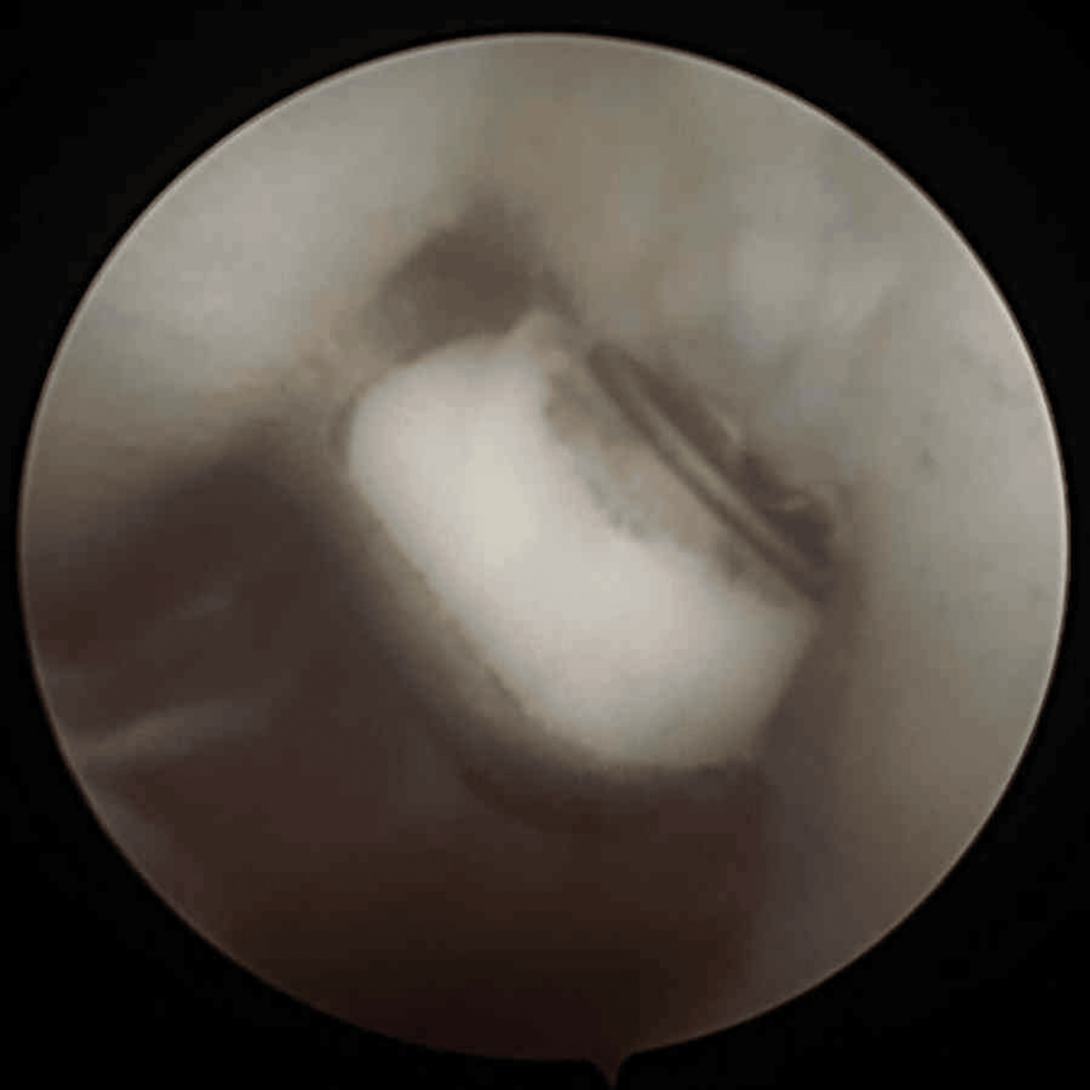
Diagnostic arthroscopy was done, which showed extensive bone loss on the glenoid and a massive Hill-Sachs lesion

**Figure 4:**
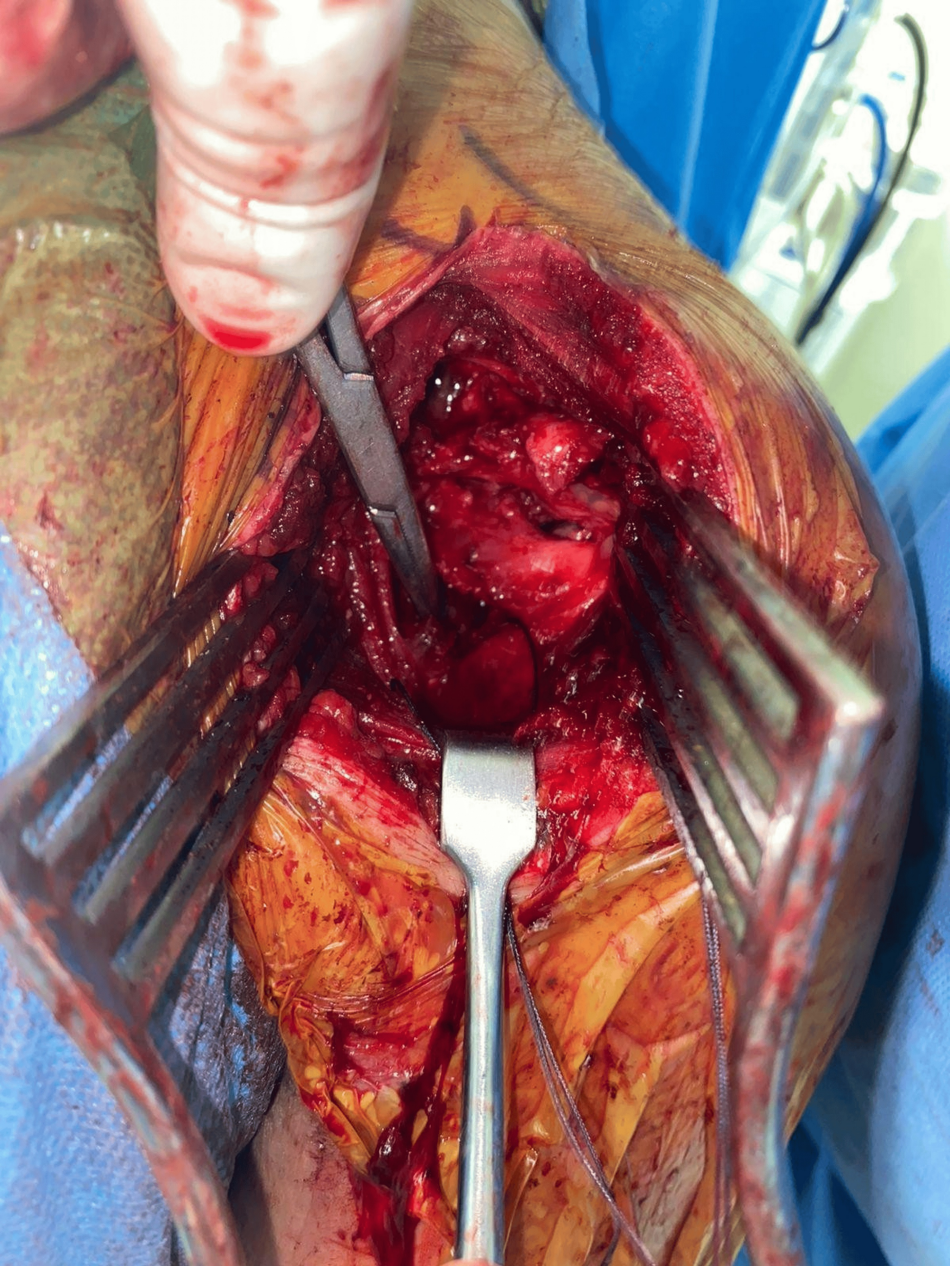
Intra-operative images

Given the extensive bone loss and engaging nature of the lesion, surgical stabilization was planned. Initially, our primary aim was to do glenoid shelving. Due to stability concerns of the involved shoulder, options were reconsidered, and it was planned for either the Eden-Hybinette procedure or a CAL. Diagnostic arthroscopy was done and confirmed extensive glenoid bone loss, a massive HSL, preserved cartilage, and a normal biceps tendon, with no loose bodies present (Figure 3). We planned for an open Latarjet procedure, as an arthroscopic Latarjet would not give the expected results. An open Latarjet procedure was performed via a deltopectoral approach. The coracoid was exposed, and the pectoralis minor was detached from its lateral side. Following coracoid osteotomy, pre-drilled holes were made in the graft. A subscapularis split was performed between the superior and middle thirds, and the anteroinferior edge of the glenoid was flattened. The coracoid graft was positioned on the anteroinferior glenoid surface and fixed with two parallel cortical screws. The capsule was closed (Figure 4).

**Figure 5:**
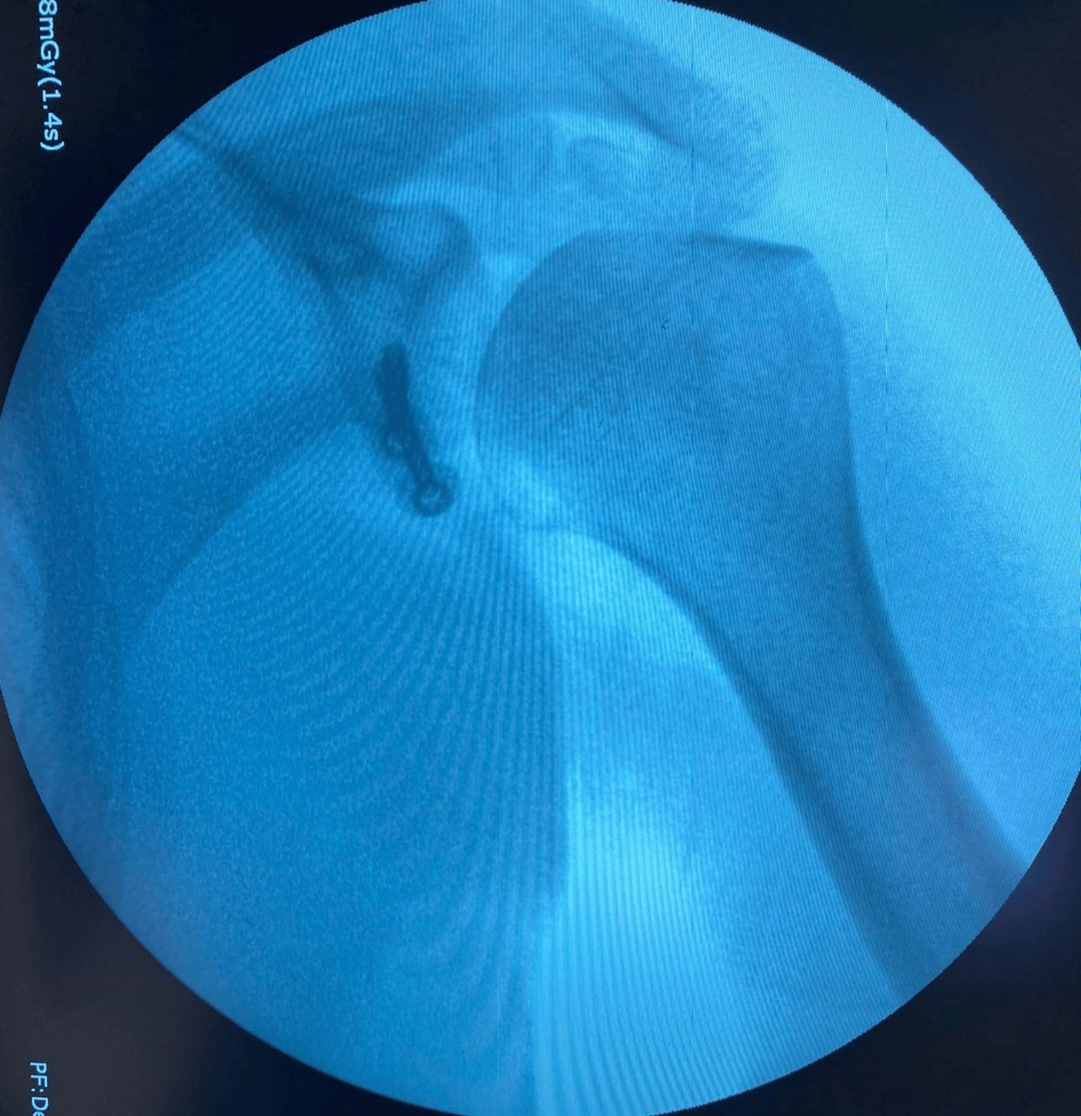
An 8C-arm image confirms the placement of two parallel screws antero-inferiorly

**Figure 6:**
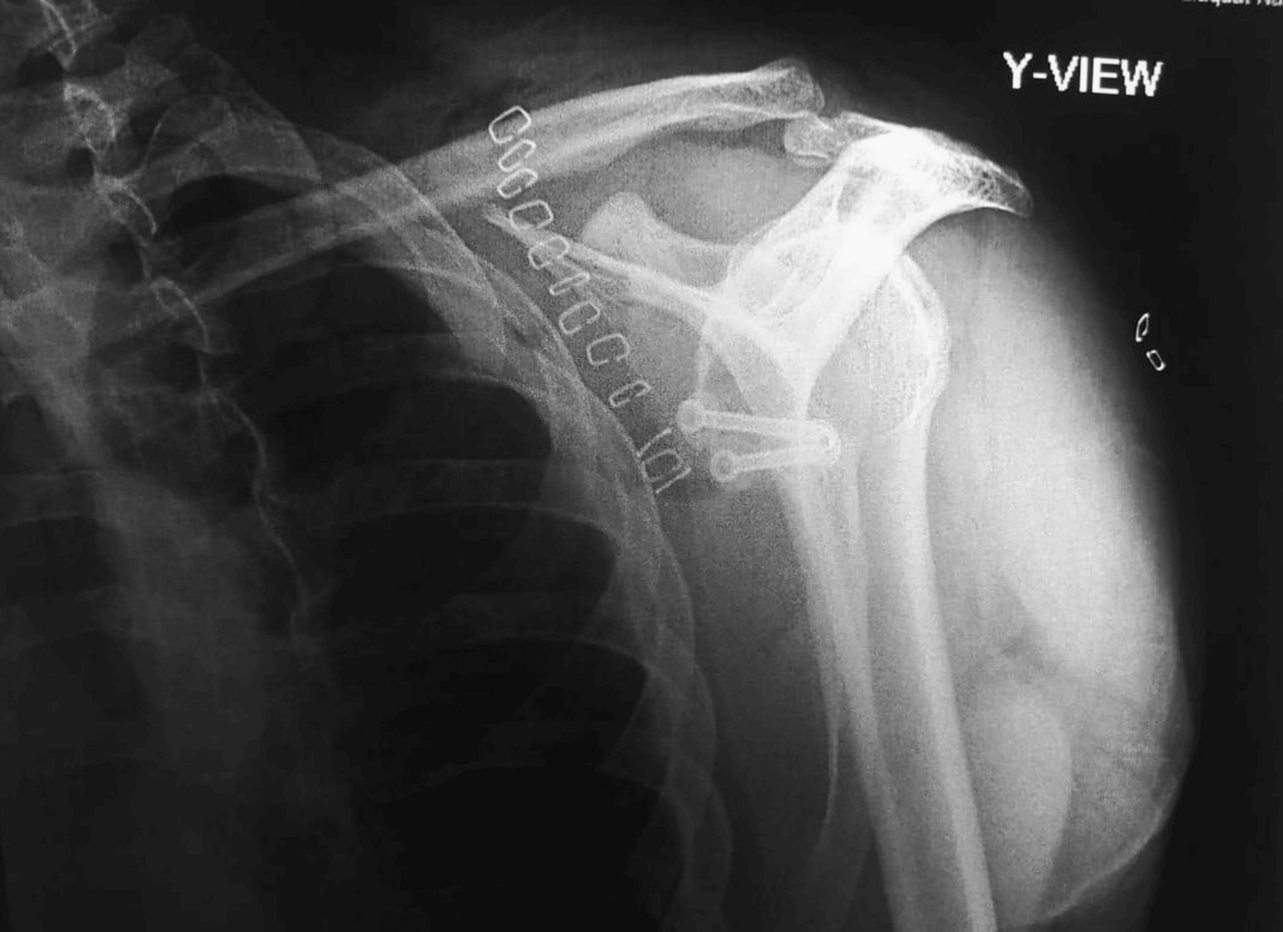
Y-view image confirms the placement of two parallel screws

Postoperatively, a radiograph confirmed the correct placement of the screws (Figures 5, 6). The patient was immobilized with a poly-sling, and shoulder range of motion was initially restricted with strict instructions to avoid abduction and external rotation. Pain control was achieved with intravenous analgesics, and intravenous antibiotics were administered. Twice-daily physiotherapy sessions were initiated after the initial immobilization phase. The patient stayed in the hospital for three to four days with no perioperative complications. At the three-month follow-up, the patient had regained a complete, pain-free range of motion in the left shoulder and was able to resume daily activities without instability or functional limitations.

## Discussion

Recurrent anterior shoulder instability is commonly linked to significant bony defects such as glenoid bone loss and HSLs. When glenoid bone loss exceeds approximately 15-20%, isolated soft tissue repair techniques like Bankart repair have a high likelihood of failure, making bony augmentation procedures essential [8]. Among these, the Latarjet procedure is one of the most established techniques because it addresses both static and dynamic stabilizing mechanisms [8]. The traditional Latarjet technique provides stability through a well-described “triple-block” effect: increasing the glenoid articular surface by transferring the coracoid graft, which augments the anteroposterior diameter; creating a dynamic sling effect with the conjoined tendon that resists anterior translation of the humeral head during abduction and external rotation; and repairing the anterior capsule to the coracoid graft for additional soft-tissue stability [9]. This combination makes it highly effective in cases of critical glenoid bone loss.

A modification known as the CAL rotates the coracoid graft by 90° so that its inferior surface, which has a greater radius of curvature, forms the new articular surface of the glenoid. This allows for better restoration of the native glenoid arc, particularly in patients with large bone defects exceeding 20% [10]. By optimizing the contour and increasing surface area, CAL aims to improve joint congruity and reduce the risk of humeral head edge-loading. A systematic review and meta-analysis comparing the traditional and CAL techniques reported that CAL achieved similar or slightly improved outcomes in terms of return-to-sport rates, recurrence, and revision surgery. However, a slightly higher rate of complications, such as fibrous union or issues with graft fixation, was observed in CAL [11]. Long-term follow-up of 136 patients who underwent CAL (mean 10 years) showed excellent results, including a 6% recurrence rate, 3% revision rate, and 92% graft union. Functional outcomes measured by Rowe, American Shoulder and Elbow Surgeons (ASES), and Single Assessment Numeric Evaluation (SANE) scores were favorable in more than 90% of patients. Mild to moderate osteoarthritis developed in 26% of cases during long-term follow-up [12].

Recent studies focusing on large glenoid defects (>21%) have shown promising results with CAL. One case series reported zero recurrences at an average follow-up of 36 months, excellent functional outcomes, and only minimal external rotation loss (~8°), which is acceptable for most active individuals and athletes [13]. These findings support the CAL modification as an effective option for high-demand patients with severe bone loss. Technical accuracy remains crucial for the success of any Latarjet procedure. Malpositioning of the graft, particularly placing it more than 1 cm medial to the glenoid rim, has been associated with recurrence rates as high as 83% [14]. To minimize complications, the graft should be placed flush or slightly medial to the glenoid rim, combined with a subscapularis split and stable screw fixation. In our case report of a young athlete with an off-track bipolar lesion and substantial glenoid bone loss, the CAL technique was selected to maximize glenoid arc restoration and stability. At 30 months postoperatively, the patient demonstrated a pain-free range of motion with no signs of instability, consistent with the positive outcomes reported in the literature for CAL.

## Conclusions

The CAL procedure is an effective solution for recurrent anterior shoulder instability with significant glenoid bone loss, especially in high-demand patients. Precise preoperative imaging, surgical planning, and accurate graft placement are critical for optimal outcomes. Our case demonstrates excellent early functional recovery and stability at three months postoperatively.
